# The effect of restorative material selection and cementation procedures on the durability of endocrowns in the anterior teeth: an in-vitro study

**DOI:** 10.1186/s12903-024-04381-9

**Published:** 2024-06-08

**Authors:** Nehal Samra, Manal M Madina, Salwa Abd El-Raof El-Negoly, Lamia Dawood

**Affiliations:** 1 Prosthodontic Department, Faculty of Dentistry, Kafr Elsheikh University, Kafr Elsheikh, Egypt; 2 Fixed Prosthodontic department, Faculty of Dentistry, Mansoura University, Mansoura, Egypt; 3 Dental Biomaterial Department, Faculty of Dentistry, Mansoura University, Mansoura, Egypt

**Keywords:** Zirconia, E-max, MDP, Fracture strength, Mode of failure

## Abstract

**Objective:**

To investigate the fracture resistance and failure modalities of anterior endocrown restorations fabricated employing diverse ceramic materials, and bonded using various cementation methodologies.

**Materials and methods:**

Forty maxillary central incisors were divided into two main groups based on the ceramic materials used; GroupI (Zir): zirconia endocrwons (Zolid HT^+^, Ceramill, Amanngirrbach) and GroupII (E-Max): e-max endocrowns (IPS e.max CAD, Ivoclar Vivadent). Both groups were further split into two subgroups depending on the cementation protocols; subgroup IA “ZirMDP”: endocowns cemented with MDP primer + MDP resin cement, subgroup IB (ZirNon-MDP): cemented with MDP primer + non-MDP resin cement, subgroup IIA (E-maxMDP): cemented with MDP primer + MDP resin cement, subgroup IIB (E-maxNon-MDP): cemented with MDP primer + non-MDP resin cement. (*n* = 10/subgroup). Endocrowns were manufactured using CAD/ CAM. Teeth were subjected to 10,000 thermal cycles. The fracture test was performed at 45^o^ with a palatal force direction until the fracture occurred. Test results were recorded in Newton. The failure mode was examined using a stereomicroscope. A One-way ANOVA test was utilized to compare different groups regarding fracture strength values. Tukey`s Post Hoc was utilized for multiple comparisons.

**Results:**

The comparative analysis of fracture strength across the diverse groups yielded non-significant differences, as indicated by a p-value exceeding 0.05. Nonetheless, an observable trend emerged regarding the mode of failure. Specifically, a statistically significant prevalence was noted in fractures localized within the endocrown/tooth complex below the cementoenamel junction (CEJ) across all groups, except for Group IIB, “E-max Non-MDP,” where fractures within the endocrown/tooth complex occurred above the CEJ.

**Conclusions:**

Combining an MDP-based primer with an MDP-based resin cement did not result in a significant effect on the anterior endocrown fracture strength.

**Clinical relevance:**

Regardless of the presence of the MDP monomer in its composition, adhesive resin cement achieved highly successful fracture strength when used with MDP-based ceramic primers. Additionally, ceramic materials exhibiting elastic moduli surpassing those of dentin are discouraged due to their propensity to induce catastrophic fractures within the tooth structure.

## Introduction

For an extended period of time, restoring endodontically treated teeth with substantial coronal damage has posed a challenge. The choice of restorative treatment is negatively impacted by the low structural integrity induced by caries and/or cavity preparation. Moreover, dehydration diminished resilience consequent to endodontic interventions pose an additional obstacle. Such factors significantly influence the biomechanical principles of resistance and retention essential for the restoration of teeth exhibiting this condition [1, 2].

Endocrown restorations float on the surface as an alternative and opponent to the traditional post and core method. Endocrowns are defined as monolithic restorations that have an anchorage extending inside the pulp chamber [3]. As reported by many authors [1, 4, 5], their superiority is attributed to the minimal removal of root dentin required for retainer installation, thereby reducing the risk of root weakening, perforation, and bacterial contamination compared to posts. Moreover, they require less preparation time and entail a reduced number of interfaces between each component of the restoration and the dental substrate [6–8].

Regrettably restorations applied to anterior teeth are subject to great bending moments as dictated by the principles of lever equilibrium. Additionally, anterior teeth possess a comparatively smaller surface area available for bonding when compared to their posterior counterparts. These distinctions render the biomechanical attributes of incisors notably more intricate and challenging [4].

The heightened emphasis on achieving naturalistic aesthetics within the esthetic zone further compounds this challenge. Consequently, the situation necessitates the selection of endocrown materials capable of withstanding substantial stress levels without compromising aesthetic appeal. Examples include zirconia and lithium disilicate, renowned for their resilience and exceptional esthetic properties [9, 10].

The advancements in CAD/CAM (Computer-assisted Designing/Computer-assisted Manufacturing) technology have facilitated the utilization of novel restorative materials, characterized by enhanced esthetics, precise fit, and marginal accuracy. Furthermore, the adoption of these materials streamlines the fabrication process of restorations in comparison to traditional preparation methods [11]. Numerous studies have highlighted zirconia’s superior fracture strength under occlusal forces when utilized for endocrowns [1, 12]. Conversely, some researchers advocate for lithium disilicate as the material of choice, citing its robust mechanical properties and superior esthetic outcomes [8, 13].

Bonding to zirconia comprises a challenge. The clinical achievement of ceramics with high strength, like zirconia, is highly dependent on the adhesion to natural tooth structure [14]. A definitive approach for optimal adhesive bonding to yttrium tetragonal zirconia polycrystals is yet to be found [15]. Several studies advocated that chemical bonds (P-O-Zr) are produced between zirconia and MDP, subsequently, when the phosphate ester monomer 10-methacryloyloxydecyl dihydrogen phosphate (MDP) was added to bonding agents the bond to zirconia seemed to become stronger [16–18].

The null hypothesis (Ho) for this study proposes that there are no significant differences in fracture strength when comparing treatment options for anterior dentition using endocrowns made from either lithium disilicate or monolithic zirconia, and subsequently cemented with adhesive resin containing MDP monomer or without it.

## Materials and methods

The protocol of this study was accepted by the Ethical Committee, Faculty of Dentistry, Mansoura University (code: M01160321/ 2021). Sample size calculated depending on a previous study (Al-Fadhli M, Mohsen C. and Katamich H (2021) Fracture resistance of anterior endocrown vs. post crown restoration an in-vitro study. Sys Rev Pharm. 12(11): 594–603) [19] as a reference. If the mean ± standard deviation of the control group is 234.74 ± 42.56, while the estimated mean of the other group is 295, with a 1.41 effect size when the power was 80% & type I error probability was 0.05. Minimally the study needed 9 subjects in each group, the total sample size was raised to 10 subjects per group to compensate 15% drop out. The sample size was carried out using the Independent t-test by utilizing G. power 3.1.9.7.

### Randomization and allocation

The study employed randomization using sequential and arbitrary assignment of unique identification numbers to ensure impartial distribution of samples. Computerized sequence generation via SPSS v. 20.0 was utilized to allocate specimens into two main groups, Zirconia (Group I) and E-Max (Group II) endocrowns. Each random number was carefully matched with the corresponding specimen number to arrange specimens accordingly. Group allocation was balanced, and each group was subdivided into two equal subgroups for specific cementation protocols utilizing the same software.

### Teeth preparation

Forty human maxillary central incisors were sourced from patients exhibiting grade III mobility due to periodontal disease, diabetes, or for prosthetic treatment purposes, with explicit consent obtained for their utilization. Inclusion criteria mandated fully formed apices, a minimum root length of 14 mm (± 0.5), straight roots, and absence of caries or fractures in the crown or root. Sterilization involved immersion in 5.25% sodium hypochlorite for 15 min at room temperature, followed by storage in 0.9% sodium chloride normal saline solution to prevent dehydration during experimentation [20]. Endodontic access was established following standardized procedures. Mechanical preparation utilized manual K-files size 20 and 25 sequentially, followed by instrumentation with a rotary endo-motor (Tri Auto mini, J. Morita GmbH, Dietzenbach, Germany) and Pro-Taper Universal files (Dentsply, Johnson City, USA). Canals were irrigated with 1% NaOCl between filing steps, and the smear layer was removed using 17% ethylenediaminetetraacetic acid (EDTA) (Md-Chelcream, Meta Biomed, Chungcheongbuk-do, Korea) followed by distilled water rinse [21]. Single cone obturation with gutta-percha (Protaper Universal, Dentsply, Johnson City, USA) and resin-based root canal sealer (Adseal, Meta Biomed, Chungcheongbuk-do, Korea) was performed, and pulp chamber access was temporarily sealed with MD-Temp Plus (Meta Biomed, Chungcheongbuk-do, Korea). Specimens were stored in saline at 37 °C and 100% humidity for 48 h to allow for cement setting [8]. A prefabricated mold facilitated fixation of teeth within standardized resin blocks with a centralizing device, leaving 1 mm between the acrylic margin and the cement-enamel junction for handling convenience [19, 22]. Anatomical crowns were marked and sectioned 2 mm above the cemento-enamel junction.

Specimens were split into two main groups based on the ceramic materials used; GroupI (Zir): zirconia endocrwons (Zolid HT^+^, Ceramill, Amanngirrbach) and GroupII (E-Max): e-max endocrowns (IPS e.max CAD, Ivoclar Vivadent). Each main group was further subdivided into two subgroups on the basis of the cementation protocols; subgroup IA “ZirMDP”: endocowns cemented with MDP primer + MDP resin cement, subgroup IB (ZirNon-MDP): cemented with MDP primer + non-MDP resin cement, subgroup IIA (E-maxMDP): cemented with MDP primer + MDP resin cement, subgroup IIB (E-maxNon-MDP): cemented with MDP primer + non-MDP resin cement. (*n* = 10/subgroup). A description of the materials used in this study and sample grouping are illustrated in in Tables 1 and 2 respectively.

**Table 1:** Materials used in this study

Material	Product Name	Composition	Manufacturer
1)Translucent Zirconia	Ceramill Zolid HT^+^	Partially stabilized with yttrium and enriched with aluminium	Ceramill Zolid HT, Amman Girrbach, Germany
2) Lithium disilicate glass ceramic	IPS e.max CAD	- Main component: SiO2 (57–80 wt%) - Other contents: Li2 O, K2 O, MgO, Al2 O3, P2 O5, ZrO2, ZnO and coloring oxides	Ivoclar Vivadent, Schaan, Liechtenstein
3) MDP resin cement	Panavia SA Cement Plus	Base: BisGMA, TEGDMA, UDMA, 10-MDP, silanized glass filler, silanized colloidal silica, photo-initiator, chemical-initiator. Catalyst: Bis-GMA, dimethacrylate, silanized Barium glass filler, silanized colloidal silica, chemical accelerator, pigment.	kuraray Noritake, Okayama, Japan
4) Non MDP resin cement	Duo-Link Universal	Base: Bisphenol-A glycidyl dimrthacrylate, uncured dimethacrylate monomer, glass filler Catalyst: phosphoric acidic monomer, glass fillers	Bisco Inc., Schaumburg, USA

**Table 2:** Sample grouping

Groups	Group I (zir): Zirconia anterior Endocrowns		Group II( E-max): E-max anterior Endocrowns	
Subgroups	**Subgroup IA (ZirMDP)** Cementation with MDP based primer+ MDP containing resin	**Subgroup IB(ZirNon-MDP)** Cementation with MDP based primer + Non MDP containing resin	**Subgroup IIA (E-maxMDP)** Cementation with MDP based primer+ MDP containing resin	**Subgroup IIB (E-maxNon-MDP** **)** Cementation with MDP based primer+ Non MDP containing resin
Number of samples per subgroup	*n* = 10	*n* = 10	*n* = 10	*n* = 10

### Endocrown tooth preparation

**Fig. 1:**
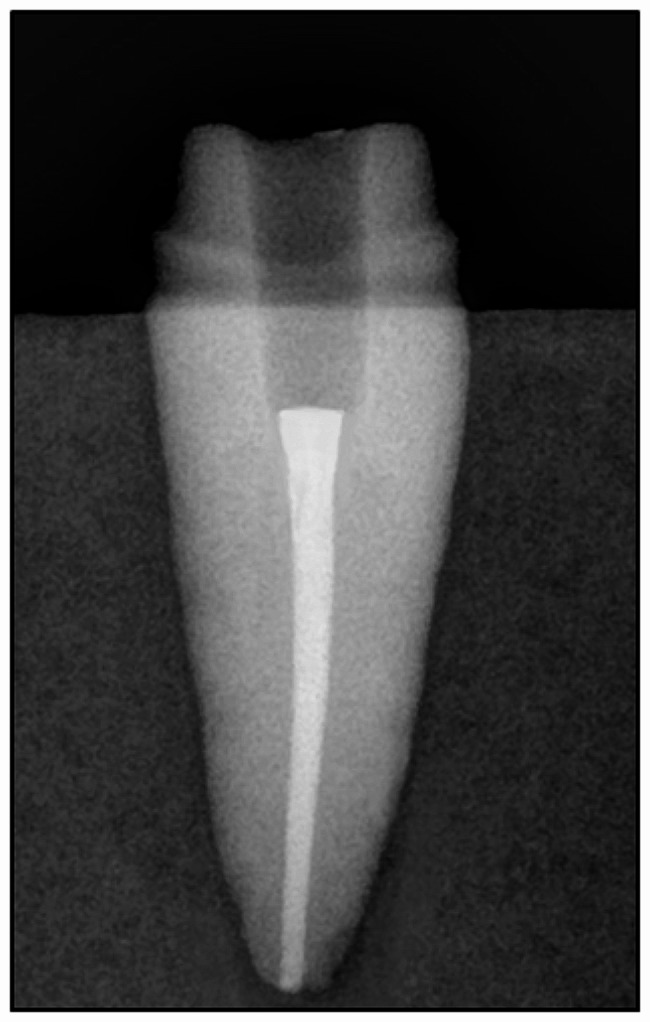
X-ray of tooth intra-radicular preparation for endocrown

**Fig. 2:**
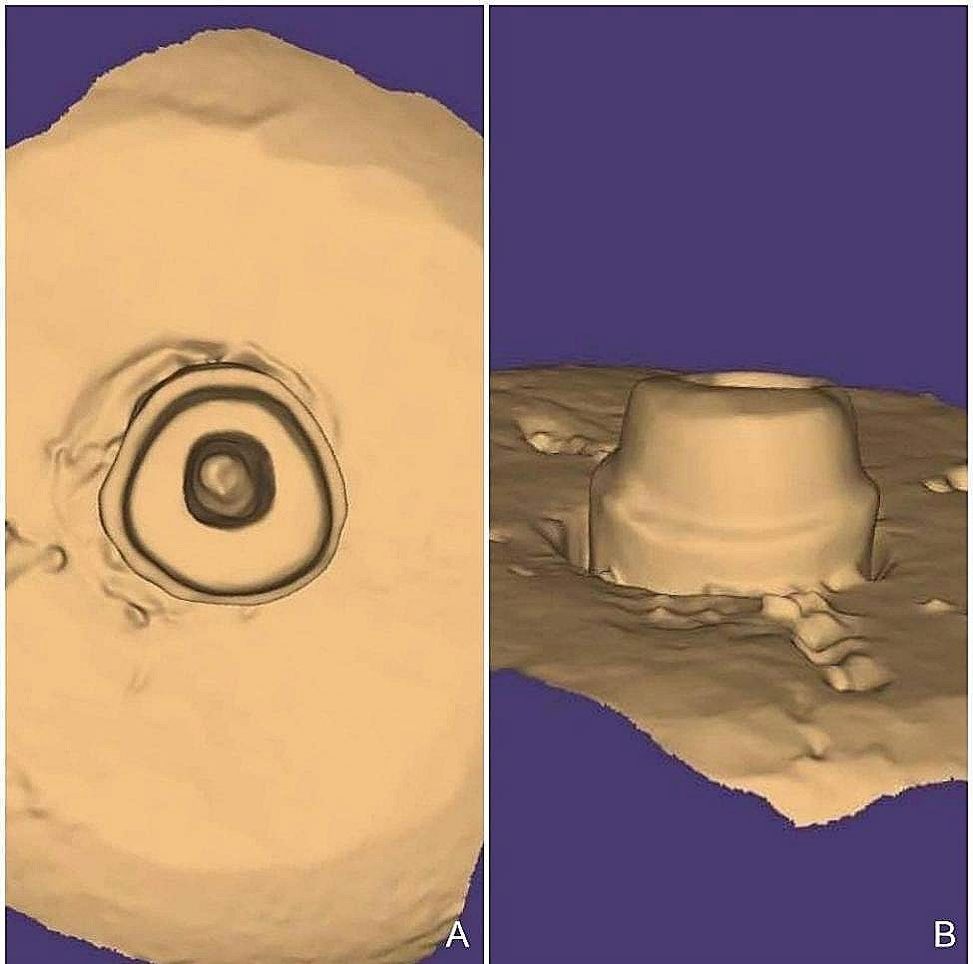
Digital scanning of the endocrown preparation. **A**: Labial view. **B**: Incisal view

The pulp chamber of all cohorts underwent preparation to eradicate any undercut areas and establish smoothly divergent walls employing diamond burs (TF 13, Mani, Tochigi, Japan) under copious water irrigation. Canals were deepened by 4 mm, with 2 mm extending from the external surface of the preparation to the base of the pulp chamber and an additional 2 mm seated within the root canal. Gates glidden drills size 2 (Gates Drills, Mani, Tochigi, Japan) were utilized to eliminate 2 mm of gutta-percha. Standardization of preparation depth was achieved using a silicone stopper. The radicular portion of the tooth was widened using diamond burs (TF 13, Mani, Tochigi, Japan) under copious water irrigation. External crown preparation entailed the use of a diamond bur (TR 13, Mani, Tochigi, Japan) held parallel to the long axis of the tooth and giving the opposing axial walls 6-8^o^ of tapering and deep chamfer finish line of 1 mm thickness [1, 8]. All preparations were conducted by the same operator, with X-rays taken to confirm intra-radicular preparation. (Fig. 1). Prepared specimens were scanned utilizing an intra-oral scanner (Medit I500, Medit Corp., Seoul, Korea). Scanning was done in accordance with the user manual’s instructions. The scanning process started with scanning the extra-coronal surface followed by the intra-coronal and intra-radicular parts ensuring that the full depth of the preparation and all details were recorded. To guarantee uniformity, the scans were conducted on the same day by the same investigator. The recorded data were processed into digital format and transferred as stereolithography (STL) files to Exocad software (DentalCAD V3.0-7662/64, Exocad GmbH, Darmstadt, Germany) (Fig. 2).

### CAD/CAM fabrication of endocrown restorations

**Fig. 3:**
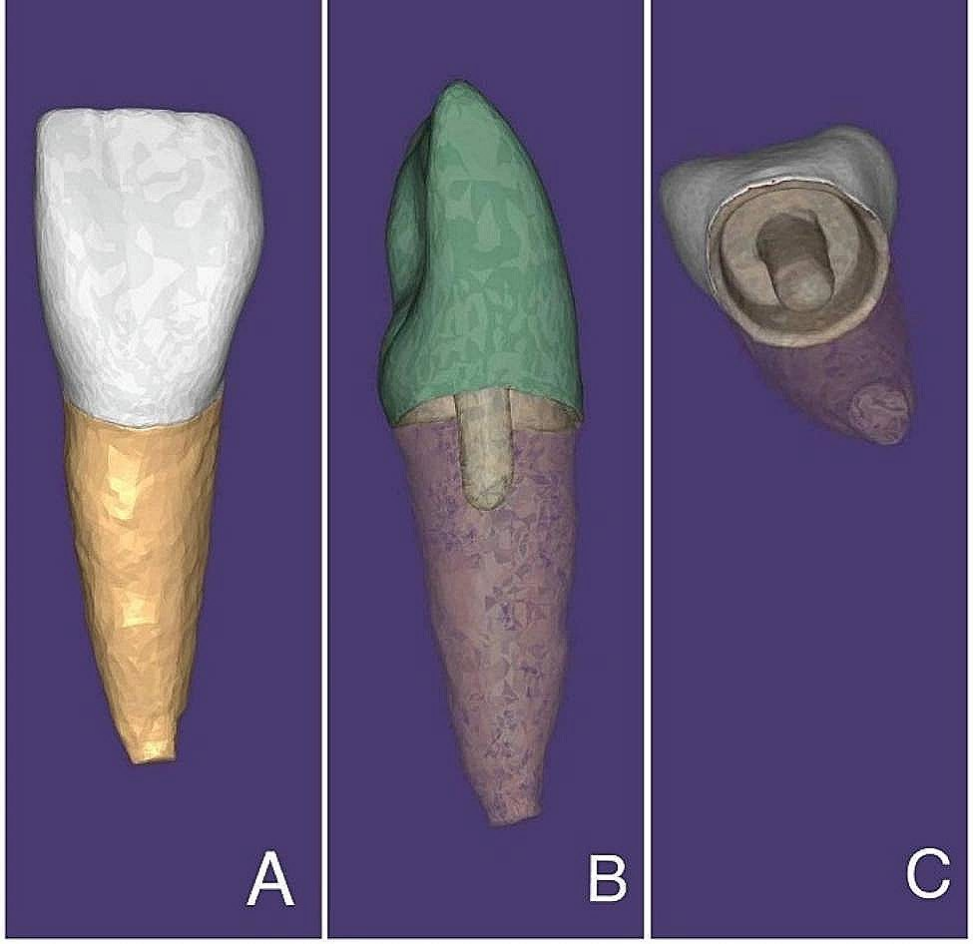
Demonstrating endocrown design from different aspects. **A**: Labial aspect. **B**: Proximal aspect. **C**: Internal aspect

To ensure uniformity across all fabricated restorations, the morphology of the maxillary right first incisor was selected from the Exocad software library (DentalCAD V3.0-7662/64, Exocad GmbH, Darmstadt, Germany). A marginal space of 20 μm was designated for the restoration, while the internal gap for the cement was set at 30 μm. A shade of A2 was uniformly selected for all restorations [22, 23]. . Examples of designed endocrown after scanning tooth preparation of an un-mounted specimen are illustrated in Fig. 3.

### Zirconia endocrown fabrication

To compensate for the sintering shrinkage, the scanned data were enlarged by 20–25%. Highly translucent pre-sintered zirconia blanks (Zolid HT^+^, Ceramill, Amanngirrbach, Koblach, Austria) were used to mill group I (Zir) of endocrowns. The dry milling mode of the 5-axis CAM dental milling machine (Core Tec 25I, Imes-Icore GmbH, Eiterfeld, Germany) was adopted. Subsequently, the milled zirconia endocrowns underwent sintering in a Tabeo furnace (Tabeo, Mihm-Vogt GmbH &Co, Stutensee, Germany).

### E-max endocrown fabrication

For group II (E-Max) endocrown, Lithium disilicate glass-ceramic blocks (IPS e.max CAD, Ivoclar Vivadent, Schaan, Liechtenstein) were utilized. Employing the wet milling mode, e-max blocks were milled using the 5-axis CAM dental milling machine (Core Tec 25I, Imes-Icore GmbH, Eiterfeld, Germany). Milled e-max endocrowns underwent crystallization firing using (Vacumat 6000 M, Vita Zahnfabrik, Bad Sackingn, Germany). All restorations were subjected to glazing procedure using paste glaze (CeraMotion Paste Glaze, Dentaurum GmbH, Ispringen, Germany).

### Endocrowns surface treatment

Following fabrication, all restorations underwent a thorough cleaning regimen, wherein they were immersed in distilled water within an ultrasonic cleaner (Ultrasonic cleaner VI, Yoshida Dental Trade Distribution Co., Tokyo, Japan) for a duration of 10 min. Subsequently, they were dried utilizing oil-free air from a triple syringe for a period of 10 s. For endocrowns within the zirconia groups, an additional step was undertaken involving air-borne particle abrasion utilizing 50 μm aluminum oxide particles (Korox 50; Bego, Bremen, Germany), directed perpendicular to the surface. This abrasion process was executed at a pressure of 2.5 bars for 10 s, maintaining a distance of 10 mm [22, 24]. .

For e-max groups, 9.5% buffered hydrofluoric acid gel (Porcelain Etchant, Bisco Inc., Schaumburg, USA) was introduced for 90 s then rinsed with copious amount of water for 10 s, followed by air drying for 5 s.

### Teeth conditioning

To prepare the teeth surfaces, etching was performed using 37% phosphoric acid (Meta Etchant, Meta Biomed, Chungcheongbuk-do, Korea) for a duration of 15 s. Subsequently, the etched surfaces were rinsed for 20 s with an air/water spray. Following this, a layer of MDP-containing bond (Tri-s bond, Kuraray Noritake, Okayama, Japan) was applied, allowed to remain for 20 s, and then light cured for 10 Sects. [25, 26].

### Cementation steps

In all experimental groups, the intaglio surface of the endocrowns received uniform application of 2 coats of MDP-based primer (Z-Prime Plus, Bisco Inc., Schaumburg, USA), after which they were subjected to air drying for a period of 5 Sects. [27, 28]. .

Endocrowns in group IA “ZirMDP” and group IIA “E-maxMDP” were cemented using MDP-containing auto-mix, dual-cure resin cement (Panavia SA Cement Plus, Kuraray Noritake, Okayama, Japan). Conversely, endocrowns in group IB “Zir Non-MDP” and group IIB “E-maxNon-MDP” were cemented utilizing non-MDP containing cement (Duo-Link Universal, Bisco Inc., Schaumburg, USA). Following cement application, excess cement was tack cured for 2–3 s per quarter surface and subsequently removed using a dental explorer. Cement setting occurred chemically over a period of 5 min in accordance with the manufacturer’s instructions. Following cementation, all specimens were stored in distilled water at 37 °C for a duration of 24 h [22]. 

### Thermocycling

All specimens underwent thermal cycling comprising 10,000 cycles using a Thermo-cycler (TC21, ROBOTA, Egypt), simulating 1 year of clinical function. The thermal cycling involved alternating immersion in water baths with temperatures ranging between 5 °C and 55 °C. Each temperature cycle consisted of a dwell time of 30 s followed by a transfer period of 10 s between baths [29]. .

### Fracture strength test and failure mode analysis

Endocrowns were exposed to fracture resistance test using a universal testing machine (Instron 3345 Instron, Norwood, United States). Each specimen was placed on a metallic device at 45^o^ to its long axis to mimic the clinical conditions. Until fracture occurred, compressive load was palatally applied at a cross-head speed of 1 mm/min. The maximum fracture load was recorded in Newton’s [8, 10].

Subsequently, specimens were examined at a magnification of ×40 under a stereomicroscope (SMZ745T, Nikon, Tokyo, Japan) to assess modes of failure. Failure patterns were classified follows: Type I: debonding, Type II: fracture of endocrown, Type III: fracture of the endocrown/tooth complex above the CEJ, and Type IV: fracture of the endocrown/tooth complex under the CEJ. Modes of failure were categorized in Table 3.

**Table 3:** Classification of the failure modes

Mode	Type	Description
Favourable	I	Debonding of endocrown
	II	Fracture of endocrown
Unfavourable	III above the CEJ	Fracture of the endocrown/tooth complex
	IV under the CEJ	Fracture of the endocrown/tooth complex

**(CEJ**: cement-enamel junction**).**

### Statistical analysis

Statistical analysis was executed utilizing SPSS 20®, Graph Pad Prism®, and Microsoft Excel 2016. Normality was confirmed by Shapiro–Wilk test. Descriptive statistics including minimum (min), maximum (max), mean (M), and standard deviation (SD) were used to summarize quantitative data. For comparing fracture strength values among different groups, One Way ANOVA test was employed, followed by Tukey’s Post Hoc test for multiple comparisons. Qualitative data were presented as frequency (N) and percentages (%), and comparisons were made using the Chi-square test.

## Results

**Fig. 4:**
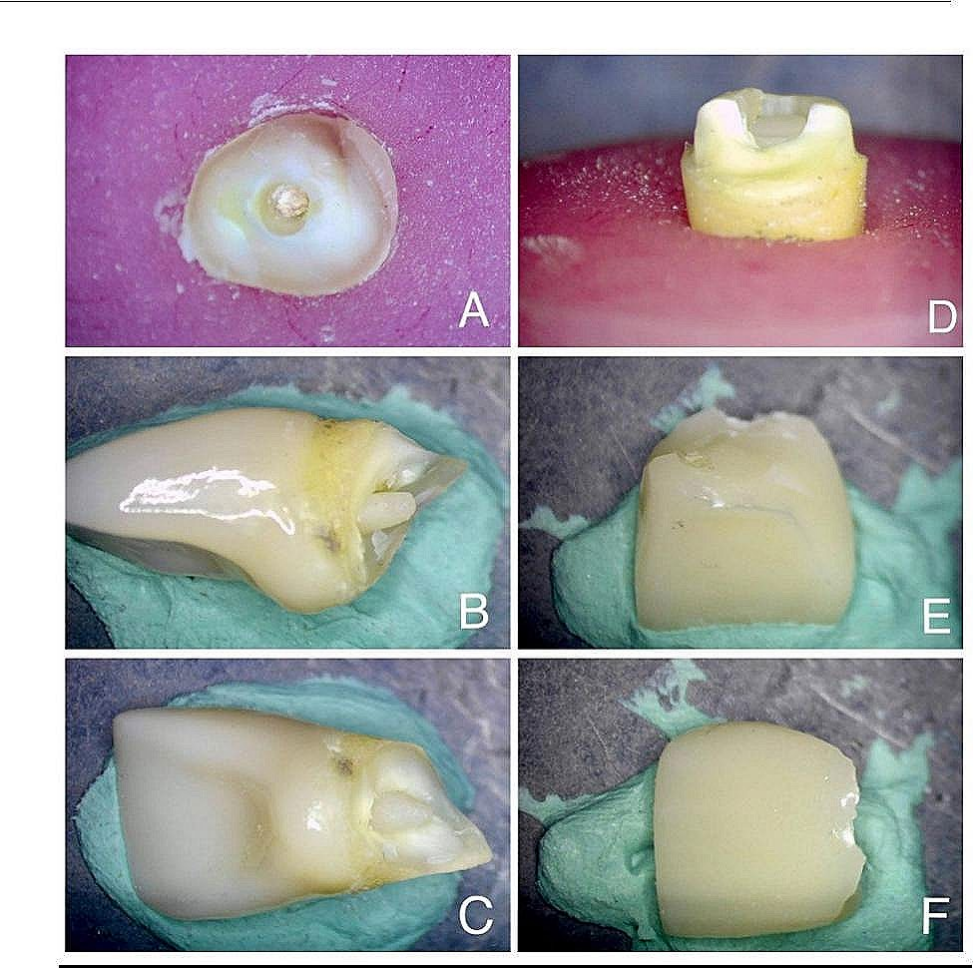
Example of the modes of failure of the specimens. **A**, **B**, and **C**: representing Type IV (unfavourable) fracture. **D**, **E**, and **F**: representing type III (favourable fracture)

The minimum (min), maximum (max), mean (M), and standard deviation (SD) of fracture strength of all groups were presented in Table 4. Group IA “ZirMDP” exhibited the lowest fracture strength with a value of 188.08 N. Group IB “ZirNon-MDP” and group IIA “E-maxMDP” recorded fracture strengths of 222.85 N and 191.79 N, respectively. On the other hand, group IIB “E-maxNon-MDP” demonstrated the highest fracture strength value at 238.06 N. Comparison among different groups using the One Way ANOVA test revealed insignificant differences between them. Table 5 displays the frequency and percentages of different types of failure observed in all groups. Analysis of the failure modes revealed that 80% of specimens in group IA “ZirMDP” and IIA “E-maxMDP” exhibited type IV unrepairable fractures, with only 20% showing type III repairable fractures. Conversely, all endocrowns in group IB “ZirNon-MDP” experienced type IV fractures. In group IIB “E-max Non-MDP”, 100% of the specimens displayed repairable fractures, with 40% classified as type II and 60% as type III. These results indicate a significant predominance of unrepairable type IV fractures across all groups. Moreover, group IIB “E-max Non-MDP” demonstrated a significant predominance of repairable fractures, particularly type III. These findings suggest a prevalent occurrence of tooth fractures either above or below the Cementoenamel Junction (CEJ) (Fig. 4).

**Table 4:** Minimum, maximum, mean, and standard deviation of fracture strength regarding all groups

Fracture strength values in *N*	*N*	Minimum (min)	Maximum (max)	Mean (M)	Standard Deviation (SD)	*P* value
IA “ZirMDP”	10	175.70	200.92	188.08	10.37	0.09 ns
IB ‘’ZirNon-MDP’’	10	217.38	230.26	222.85	5.64	
IIA ‘’E-maxMDP’’	10	103.76	268.24	191.79	79.69	
IIB ‘’E-maxNon-MDP’’	10	180.21	309.09	238.06	61.62	

**Ns**: non-significant difference as *P* > 0.05

**Table 5:** Frequency and percentages of different scores in all groups and comparison between them using Chi-Square test

Groups	Type I		Type II		Type III		Type IV	
	n	%	n	%	n	%	n	%
IA *“ZirMDP”*	0	0	0	0	2	20	8	80
IB *‘’ZirNon-MDP’’*	0	0	0	0	0	0	10	100
IIA *‘’E-max MDP’’*	0	0	0	0	2	20	8	80
IIB *‘’E-max Non-MDP’’*	0	0	4	40	6	60	0	0

## Discussion

This study sought to investigate whether there exists a significant distinction in fracture strength between e-max and zirconia materials when employed for endocrown fabrication in anterior teeth treatment, even under diverse cementation modalities. The null hypothesis was accepted based on the findings of the One-Way ANOVA test, which revealed no statistically significant results among the examined groups.

The Yttrium-oxide-partially stabilized zirconia (Y-TZP) is documented in the literature as a reliable dental restorative material, demonstrating a notably high clinical success rate. The use of monolithic zirconia restorations presents advantages in terms of elevated fracture toughness and reduced incidence of ceramic breakage [30, 31], while concurrently offering aesthetic appeal. Consequently, there has been a surge in the utilization of Y-TZP as a fundamental material for the restoration of anterior teeth [32, 33].

Lithium disilicate glass-ceramics have become essential in dental aesthetics due to their exceptional transparency and customizable color options. Their unique interlocking structure of a glass matrix and a crystalline phase, combined with low thermal expansion and high flexural strength, prevents microcrack propagation, enhancing both structural integrity and aesthetic performance. Consequently, they are highly regarded for endocrown restorations [34, 35].

This research specifically tested the effect of combining MDP-based primer with MDP- based adhesive resin cement on the fracture strength of anterior endocrown restorations manufactured with zirconia and e-max ceramics. On one hand, both groups used MDP ceramic primer, but on the other hand, the groups (IB, IIB) used non-MDP containing resin cement, while the groups (IA, IIA) utilized resin cement with MDP functional monomer.

Several studies [14, 36] have demonstrated that utilizing a primer containing 10-methacryloyloxydecyl dihydrogen phosphate (MDP) significantly improves bonding efficacy to zirconia. This enhancement is attributed to MDP’s adsorption onto the zirconia surface layer, reinforcing chemical interaction. Additionally, researchers [37, 38] have suggested that MDP’s vinyl group facilitates polymerization interaction within the resin matrix, while its phosphoric acid group promotes adhesion with metal oxides like zirconia and alumina. Furthermore, the inclusion of bifunctional silane molecules, such as MDP, in adhesive systems applied to glass ceramics like e-max, promotes copolymerization with composite resin, forming a siloxane network on ceramic surfaces and enhancing adhesion strength [39–41].

In the literature, combining both MDP-based primer with MDP containing resin cements was believed to have a strengthening effect on bonding endocrowns to the tooth structure and subsequently adds to the strength of the tooth/ adhesive resin cement/restoration complex. It is plausible to anticipate that the MDP monomer present in the luting cement may undergo recharging with a primed layer even subsequent to solvent volatilization. Consequently, the experimental group containing MDP within both the primer and the luting resin cement would likely offer an increased provision of dihydrogen functional groups. These functional groups possess the capability to engage in chemical bonding with ceramics to a greater extent compared to the experimental group containing MDP solely within either the primer or luting cement [42–44].

In order to assess the potential impact of aging components on the durability and success of the restorations, intra-oral conditions were simulated in vitro. Endocrowns underwent 10,000 alternative hot-cold cycles in water baths to mimic intra-oral thermal variations. The temperature ranged between 5 and 55 °C with a dwell time of 30 s per cycle. According to previous studies [22, 45], such thermal cycle tests induce alternating stresses at material interfaces due to temperature fluctuations. Moreover, the disparate coefficients of thermal expansion between materials may lead to adhesive failure under varying temperatures. The number of thermal cycles used across numerous investigations [45–47] varied from 1 to 1,000,000 cycles, with an average of approximately 10,000 cycles, aiming to emulate one year of clinical service, as adopted in this study.

Given that assessing the performance and longevity of restorations involves considerations beyond the impact of thermal cycles alone, such as their ability to withstand masticatory load [10, 19], specimens were subsequently subjected to a fracture resistance test. This test aimed to evaluate the maximum capacity of the specimens to withstand occlusal forces. Researchers recommended applying a compressive force directed palatally at 45^o^ to simulate the direction of incisal forces [1, 8, 10, 48].

Results of this study showed that group IIB ‘’E-maxNon-MDP’’ exhibited the highest fracture strength value (217.38 N) whereas group IA “ZirMDP” manifested the lowest fracture strength values (103.76 N). Moreover, statistical analysis did not detect any significant differences among the various groups.

Regarding the utilization of MDP-based resin cement, the findings of this study align with the conclusions drawn by Almaskin et al. [49] and Go et al. [50]. They observed that non-MDP-containing adhesive resin cements exhibited enhanced bonding values when used in conjunction with MDP-based primers for zirconia ceramics. However, the incorporation of MDP in both the resin cement and ceramic primer did not significantly strengthen the adhesion bond. Similarly, Zhao et al. [18] noted that supplementing MDP in the cement alongside its presence in the ceramic primer did not yield any advantageous effects on durable bonding to zirconia.

The present study’s findings diverge from those of prior investigations [42–44], which advocated for the use of MDP-based adhesive resin cement alongside MDP-containing primer to achieve durable adhesion. Discrepancies in results may be attributed to variations in surface treatment techniques. For example, Lim et al. [43] not only applied MDP monomer to the zirconia surface but also to the dentine surface, potentially impacting the outcome significantly. Additionally, Salama and Salem [42], as well as Xiong et al. [44], reported conflicting results, likely due to the utilization of different combinations of adhesive monomers and resin cements from that used in this study. This suggests that factors beyond the MDP monomer, such as distinct chemical compositions of primers and cements, may play a significant role in influencing bond strength.

Examining the fracture patterns of the test specimens is thought to give a great interpretation of the future performance of the restoration intra-orally. This was crucial since failure patterns revealed if the various materials and tooth structure could be restored. After the fracture resistance test, all specimens were checked using a stereomicroscope (SMZ745T, Nikon, Tokyo, Japan) to investigate the mode of failure [8, 48, 51]. 

The failure modes were classified as repairable (Type I, II, III) and unrepairable (Type IV) according to previous literature [1, 10]. Our findings indicated a predominance of Type III and Type IV fractures, observed either above or below the CEJ, which can be attributed to the reduced number of interfaces associated with the endocrown design. This minimized adhesive interface failure [8, 48].

Furthermore, the substantial strength of the adhesive layer formed by Panavia SA and Duo-Link cements, particularly when combined with the MDP ceramic primer, significantly contributed to the formation of this robust bond and consequently, the prevalence of catastrophic failure [19, 48].

These findings are congruent with those reported by Kanat-Ertürk et al. [1]. Their study indicated that failures resulting in tooth fractures were predominantly observed in materials exhibiting higher elasticity moduli than dentin (18.6 GPa). Given the significantly higher moduli of e-max and zirconia materials used in our study (95 GPa and 200 GPa respectively) [30, 52], it is probable that the majority of endocrowns fabricated from these materials experienced failure modes characterized by catastrophic tooth fracture, specifically Type III and Type IV.

Furthermore, Güngör et al. [8] conducted a study comparing the fracture modes of post and core-restored endodontically treated teeth to endocrowns, both cemented with dual-cure resin cement (Panavia F2.0, Kuraray), and confirmed similar outcomes. They observed that while the dislodgment mode of failure predominated in the post group, endocrowns exhibited significant failure modes characterized by tooth fracture. This finding was corroborated by Silva-Sousa et al. [10], whose analysis also revealed a notable prevalence of Type III and IV failures in e-max endocrown groups.

In contrast, findings from the study conducted by Badr et al. [48] yielded divergent results. They observed that the majority of failures across all endocrown groups were repairable. This outcome was attributed to the use of nano-ceramic resin material exclusively in fabricating the endocrown restorations. In line with previous theories posited by [1, 8, 10], the close proximity of the material’s elastic modulus to that of dentin, coupled with the application of a thick layer of resin cement, acted as a stress absorber. Consequently, this mitigated the forces exerted on the root, resulting in a predominance of repairable fracture types.

## Conclusions

Within the limitation of this study, the following was concluded:

- Combining both primer and resin cement containing MDP monomer did not significantly affect the fracture strength of zirconia or e-max endocrowns.
- The use of ceramic materials with elastic moduli higher than dentin resulted in tooth catastrophic fractures.

This article used human extracted teeth. Teeth were extracted due to grade III mobility or in the favour of pre-planned prosthetic treatment with a written informed consent in accordance with the Declaration of Helsinki.

## Data Availability

The datasets used and/or analyzed during the current study are available from the corresponding author upon reasonable request.
